# Editorial: Influence of psycho-emotional factors on motor control: cerebral mechanism and behavioral response underlying (motiv)action

**DOI:** 10.3389/fnhum.2025.1572614

**Published:** 2025-03-06

**Authors:** Guillaume Léonard, Pierre-Paul Vidal, Thierry Lelard

**Affiliations:** ^1^Research Centre on Aging, Centre intégré universitaire de santé et de services sociaux de l'Estrie — Centre hospitalier universitaire de Sherbrooke, Sherbrooke, QC, Canada; ^2^School of Rehabilitation, Faculty of Medicine and Health Sciences, Université de Sherbrooke, Sherbrooke, QC, Canada; ^3^Centre Giovanni Alfonso Borelli, Université de Paris - Ecole Normale Supérieure Saclay - Centre National de la Recherche Scientifique - SSA, UMR 9010, Paris, France; ^4^Plateforme d'Etude de la Sensorimotricité, Centre National de la Recherche Scientifique UAR2009, Université Paris Cité, Paris, France; ^5^Institute of Information and Control, Hangzhou Dianzi University, Hangzhou, China; ^6^UR-UPJV EA 3300, APERE - Adaptations Physiologiques á l'Exercice et Réadaptation á l'effort - UFR des Sciences du Sport, Université de Picardie Jules Verne, Amiens, France

**Keywords:** motor control, behavioral adaptations, motor system, emotion, mental states, motivation, cognition

Movement performance can be influenced both by internal and external factors that can affect motor control. The articles featured in this Research Topic illustrate these influences, focusing on how motivational components and psycho-emotional states—driven internally by individual characteristics or externally by environmental conditions—can impact motor control.

In one of these articles, Felippin et al. examined the concept of affordance, which refers to the action possibilities an environment provides, determined by an individual's abilities, skills, or perception of their capacity to interact with a specific object. To explore this concept, the authors designed a simple albeit elegant protocol in which participants assessed how various objects should be grasped. These assessments were compared according to the emotional categories of the objects (pleasant, unpleasant, and neutral) while accounting for the influence of object size. Their results revealed that unpleasant objects were more likely to be judged as needing to be grasped accurately than pleasant or neutral objects—an effect that was consistent for both small and large items. These findings suggest that the emotional value attributed to objects can affect affordance judgments, favoring cautious handling and limited physical contact with unpleasant object, regardless of object size.

For their part, Pierrieau et al. looked at the effects of affective emotional images on neural control mechanisms. The researchers used well established protocols—based on the International Affective Picture System and on reaching paradigms—to connect affective neuroscience with motor control. Intermuscular coherence (IMC) between the electromyographic (EMG) activity of muscle pairs was used to assess changes in muscle activation patterns during reaching movements toward positive, negative, and neutral pictures, and to evaluate the impact of these stimuli on neural control mechanisms. IMC is founded on the concept of muscle synergy, which postulates that motoneurons of synergistic muscles share common corticospinal drives, leading to intermuscular coupling that can be quantified by measuring EMG oscillatory synchronicity in synergistic muscle pairs (Farmer et al., 2007). Analyzing IMC indices thus allow for the exploration of neural drive patterns that govern intermuscular coordination, providing a useful framework for understanding how viewing images with different emotional charges can influence the motor commands issued by the central nervous system (Kattla and Lowery, 2010). With regard to kinematics, the authors observed that hand movement time was greater for unpleasant pictures than for pleasant ones. Additionally, and perhaps most notably, the authors noted a reduction in IMC indices among the recorded postural muscle pairs during the initiation of pointing movements prompted by unpleasant images. Altogether, these findings provide new insights into the link between emotional contexts and motor performance, revealing that emotional stimuli can significantly impact the motor commands relayed by the central nervous system during voluntary goal-directed movements.

While the two first articles focus on how emotional information from the environment can modulate voluntary grasping or pointing movements, Phantanourak et al. investigated how individuals adapt to situations that threaten their balance. Postural threat paradigms have been widely used to study the effects of anxiety and fear of falling on movement. These studies have revealed behavioral adaptations, such as freezing or adaptation of anticipatory of postural adjustments (APAs) when standing elevated at different heights from the ground. The aim of Phantanourak et al.'s study was to determine whether the adaptation of APAs to the different levels of forces (between 50% and 90% MVC) could be modified by aging or by changes in emotional state elicited by postural threat (Carpenter et al., 2001, 2004; Sturnieks et al., 2012). To this end, the authors used an experimental task which consisted of pulling a handle attached to a strain-gauge transducer. As expected, the results indicate that young adults and, to a lesser extent, older adults adapt their APA to the force applied, with larger forces being preceded by larger and earlier APA. Importantly, APA adaptability was reduced in older adults, who show smaller increases in center of pressure (COP) velocity and in muscle activity with increasing force. An original aspect of this study lies in the apprehension created by the possibility that the platform on which the participants were standing could move at any moment, inducing apprehensiveness regarding balance. As expected, the presence of a postural threat increased physiological arousal, perceived anxiety and fear of falling, while reducing balance confidence. These changes in emotional state and physiological arousal were associated with changes in APA. Regardless of force, both young and older adults reduced their COP displacement in the presence of postural threat.

Finally, Guan et al.'s narrative review allow us to better understand the effects of regular physical activity on major depressive disorder (MDD). While the scientific literature clearly demonstrates that physical activity can improve mood and mental health in patients with MDD, the relationship between dosage of physical activity and its physiological and psychological effects remains to be clarified. After a brief overview of the clinical symptoms and pathophysiology of MDD, the narrative review explores the effects of different physical activity programs. The specific aim of this review was to highlight the influence of physical activity doses (endurance training, explosive interval training, strength resistance training, and mind body exercises) on MDD symptoms, executive functions, and sleep. In addition to outlining the effects of physical activity on key functions, the authors also highlighted the biological effects of physical activity and the mechanisms that may contribute to symptom improvement in patients with MDD as structural and functional changes in the nervous system.

Taken together, these studies provide insights into how emotional information can influence movement. These research topics can be addressed at different levels, from a subjective, behavioral, and neurophysiological perspective. Interestingly, the link between emotion and motor control is bidirectional, as nicely exposed by the narrative review looking into the effects of physical activity on mental health and depressive symptoms. Clearly, the neural mechanisms linking movement and emotion are complex and represent a vast and exciting field of research, with potential benefits for rehabilitation, ergonomics, and sports performance. The articles in this Research Topic provide just a few examples of these potential benefits and of the opportunities we have to advance knowledge in this field.
